# A Novel Self-Adaptive Control Method for Plasma Electrolytic Oxidation Processing of Aluminum Alloys

**DOI:** 10.3390/ma12172744

**Published:** 2019-08-27

**Authors:** Kai Yang, Jiaquan Zeng, Haisong Huang, Jiadui Chen, Biao Cao

**Affiliations:** 1 Key Laboratory of Advanced Manufacturing Technology of the Ministry of Education, Guizhou University, Guiyang 550025, China; 2 College of Mechanical and Automotive Engineering, South China University of Technology, Guangzhou 510640, China

**Keywords:** plasma electrolytic oxidation, electrical characteristic, anodizing, oxidation, aluminum

## Abstract

Plasma electrolytic oxidation processing is a novel promising surface modification approach for various materials. However, its large-scale application is still restricted, mainly due to the problem of high energy consumption of the plasma electrolytic oxidation processing. In order to solve this problem, a novel intelligent self-adaptive control technology based on real-time active diagnostics and on the precision adjustment of the process parameters was developed. Both the electrical characteristics of the plasma electrolytic oxidation process and the microstructure of the coating were investigated. During the plasma electrolytic oxidation process, the discharges are maintained in the soft-sparking regime and the coating exhibits a good uniformity and compactness. A total specific energy consumption of 1.8 kW h m^−2^ μm^−1^ was achieved by using such self-adaptive plasma electrolytic oxidation processing on pre-anodized 6061 aluminum alloy samples.

## 1. Introduction

Plasma electrolytic oxidation processing allows the creation of durable, thick, uniform and strongly adherent coatings on valve metals [1,2]. Extensive investigations on plasma electrolytic oxidation have been carried out in past years, due to its increasing use in industrial applications. Most of the research focuses on processing conditions [3,4,5,6,7,8], discharge characteristics [1,9,10,11,12,13], coating microstructure [14,15,16], mechanical properties [17], environmental performance [16,18], and functional characteristics [2,18]. As a result, the correlations [3,4,5,13,14,15,19] between the electrical conditions, the electrolyte compositions, the coating microstructure, and the growth rate of coating, which are linked via the characteristics of the discharges, have become clearer over recent years. Moreover, progress has been made [20,21,22,23,24,25] in unraveling the inter-relation between the energetics of individual discharges, the pulse energy, the soft-sparking regime, and the energy consumption.

Recently, the main research focus has been on the dielectric breakdown of oxide films and on the associated discharges that repeatedly occur over the whole surface of the metal substrate. It is clear that most of the new oxide of the coating created during each discharge is formed within the plasma as it cools and collapses [1,5,26]. Although the nature of the plasma created via the discharges, which occur during the plasma electrolytic oxidation process, is still uncertain, it is clear that the discharge characteristics are affected by a series of electrical conditions. Hence, there is considerable scope for more effective process electrical control, with specific objectives in terms of coating performance and energy efficiency, and an attempt is made to identify key points that are likely to assist this.

Several common electrical conditions, such as the use of direct current (DC), alternating current (AC), unipolar, and bipolar pulses, have been employed over recent years. It has been repeatedly found that the AC mode can provide more effective processing and higher quality coatings [1,27]. Furthermore, square waveforms and higher frequencies are being increasingly exploited in research and in commercial use, due to their more sophisticated impulse energy control capabilities [28,29,30]. However, the most common control modes at the basis of the plasma electrolytic oxidation processing can be classified as current-control and voltage-control. Meanwhile, most of the electrical parameters, which are applied in practice, are fixed or are chosen within a range of preset values on a largely empirical basis [31]. The major disadvantage of such control modes is that the applied electrical parameters cannot be adjusted automatically to match the dynamic requirements of the plasma electrolytic oxidation coating during its growth process.

Furthermore, high energy consumption restricts the performance of traditional plasma electrolytic oxidation processing; by exposing a metal substrate to oxidizing agents and highly energetic discharges, in fact, its discharge mechanism is extremely inefficient. Troughton et al. explored the energies absorbed by various phenomena taking place during a plasma electrolytic oxidation process (melting and vaporization of substrate, melting of existing oxide coating, initiation and sustaining of the plasma, vaporization of water, and electrical heating of the electrolyte) and inferred that most of the injected energy is absorbed in the form of the vaporization of water [32]. What is more, there are several studies [1,24,25] aimed at high energy efficiency and helpful measures. The most promising approach is probably to somehow reduce the energy associated with each discharge, possibly by reducing the voltage needed for it to occur, or to promote more transformation per discharge. Therefore, it is urgent to develop an energy-efficient intelligent process control method.

In this study, a novel self-adaptive control method based on a real-time diagnostic and a precision pulse energy regulation technique was employed for the plasma electrolytic oxidation processing of 6061 aluminum alloys. Moreover, the electrical characteristics, coating microstructure, and energy consumption of such processes were investigated.

## 2. Materials and Methods

### 2.1. Materials

A quantity of 6061 aluminum alloy samples with dimensions of 60 mm (L) × 60 mm (W) × 2 mm (H) and a 2 L water-cooled stainless steel tank, which served as a counter-electrode, were used. KOH (5 g L^−1^) and Na_2_SiO_3_ (10 g L^−1^) were dissolved in distilled water to form the electrolyte. The plasma electrolytic oxidation process was performed by using a home-built 20 kW unipolar pulsed power supply, working in voltage-control mode. The supply could provide a maximum voltage of 600 V and a maximum frequency of 10 kHz. The temperature of the setup was maintained at 25 °C.

### 2.2. Self-Adaptive Control Method

**Figure 1:**
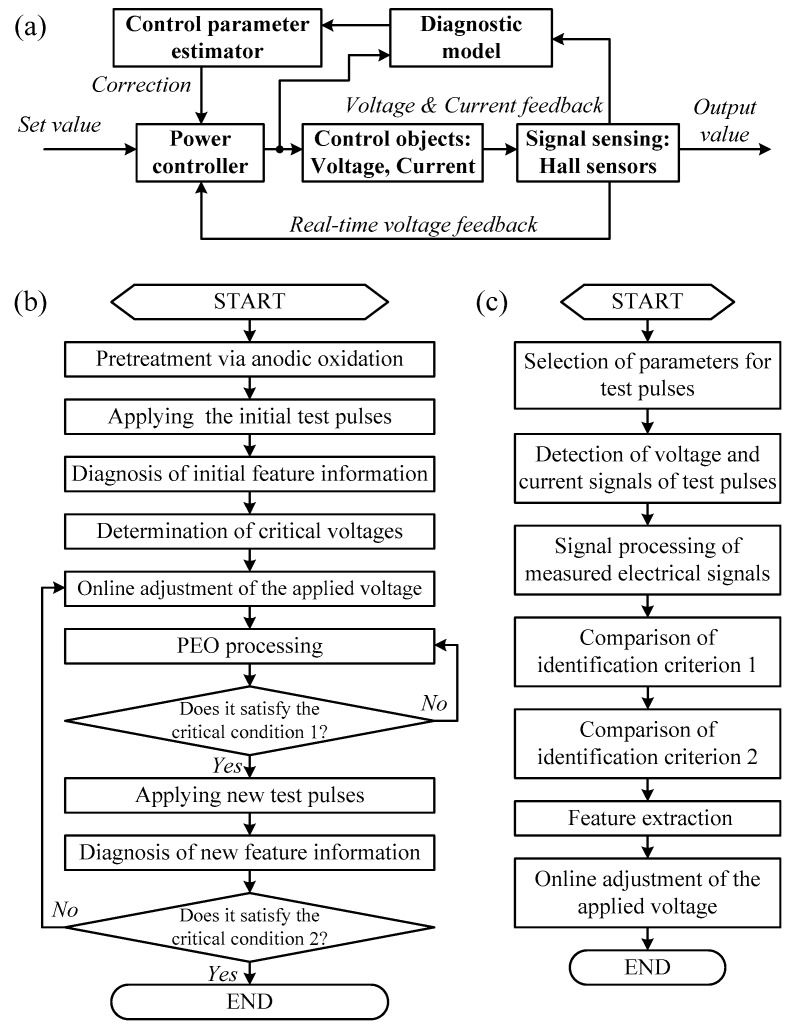
Process control method: (**a**) Self-adaptive control model, (**b**) self-adaptive control process, and (**c**) feature information identification algorithm.

The applied self-adaptive control model was operated in a double-closed loop (Figure 1a). The voltage loop ensured a constant-voltage control, whereas the voltage–current loop ensured the online adjustment of the process parameters via an active diagnostic method. The complete workflow of the self-adaptive plasma electrolytic oxidation process investigated in this paper is presented in Figure 1b. Initially, the samples were anodized up to 300 V at a rate of 20 V/min. Successively, initial test pulses were applied to determine the initial breakdown voltage (*V_i_*) and the termination voltage (*Vu*) of the sample. These parameters are listed in Table 1 (type 1). Finally, the plasma electrolytic oxidation processing was implemented by using the self-adaptive unipolar voltage pulsed mode (*f* = 100 Hz, *d* = 50%). The applied voltage was dynamically adjusted by using Equation (1):

(1) $$U=V_{b}+\Delta U.$$

Here, *U* is the voltage applied to the sample, *V_b_* corresponds to the breakdown voltage, and Δ*U* represents the voltage deviator (0–5 V).

The processing times to maintain the same applied voltage magnitude and for completing the total process were determined by using the critical condition 1 (Equation (2)) and 2 (Equation (3)), respectively.

(2) $$I\leq \frac{1}{2}I_{b}.$$

(3) $$U\geq V_{u}.$$

In Equations (2) and (3), *I* and *U* represent the real-time feedback current and the voltage, whereas *I_b_* corresponds to the breakdown current.

The key identification algorithm to obtain the coating feature information is shown in Figure 1c. The parameters of the test pulses acquired during the plasma electrolytic oxidation process are listed in Table 1 (type 2). The identification criterion 1 was used to determine the dynamic values of *V_b_* and *I_b_* as follows:

$$\mathrm{If}\;{\left(\frac{di}{dt}\right)}_{n}\approx 0\;\mathrm{and}\;{\left(\frac{di}{dt}\right)}_{n+1}\geq 1,\;\mathrm{then}\;V_{b}=V_{n},\;I_{b}=I_{n}.$$

The identification criterion 2 was used to determine the dynamic values of *V_u_* as follows:

$$\mathrm{If}\;{\left(\frac{di}{dt}\right)}_{n}\geq 0,\;{\left(\frac{di}{dt}\right)}_{n+1}\leq 0,\;\mathrm{and}\;(I_{n}>I_{b}),\;\mathrm{then}\;V_{u}=V_{n},$$

where, ${\left(\frac{di}{dt}\right)}_{n}$ is the rate of current change of pulse n, ${\left(\frac{di}{dt}\right)}_{n+1}$ is the rate of current change of pulse *n* + 1, *V_n_* represents the voltage magnitude of pulse n, and *I*_n_ represents the current magnitude of pulse n.

A detailed description of the pulse test method and of the feature information extraction can be found in a previous work [6].

### 2.3. Data Monitoring and Pre-Processing

The voltage and the current data were detected by using a hall voltage sensor (CHV-25P) and a hall current sensor (CSM010B), and recorded with a sampling frequency of 1 MHz via a data acquisition card NI PCI-6133 controlled by LabVIEW software.

Considering the signal interference factors that exist in the plasma electrolytic oxidation process, such as mechanical vibration, electron avalanche, dielectric breakdown, and high-frequency electronic switching, a pre-processing of raw voltage and current data was carried out. The data were first filtered with a low-pass filter with a cut-off frequency of 50 kHz. This allowed high-frequency noise due to electronic switching to be eliminated. Next, these data were smoothed using a moving average filter. Finally, an appropriate scale function and wavelet basis function were adopted to extract a random noise signal.

### 2.4. Post-Processing of Samples

The surface morphology, the cross-section, and the chemical composition of the coatings prepared on the samples were observed by employing a LEO1530 VP scanning electron microscope (SEM) equipped with an X-ray energy dispersive spectroscopy (EDS) setup. The phase of the coating was analyzed via X-ray diffraction (XRD, Model D8 Advance) by using a Cu kα radiation source.

## 3. Results

### 3.1. Electrical Characteristics of the Plasma Electrolytic Oxidation Process

**Figure 2:**
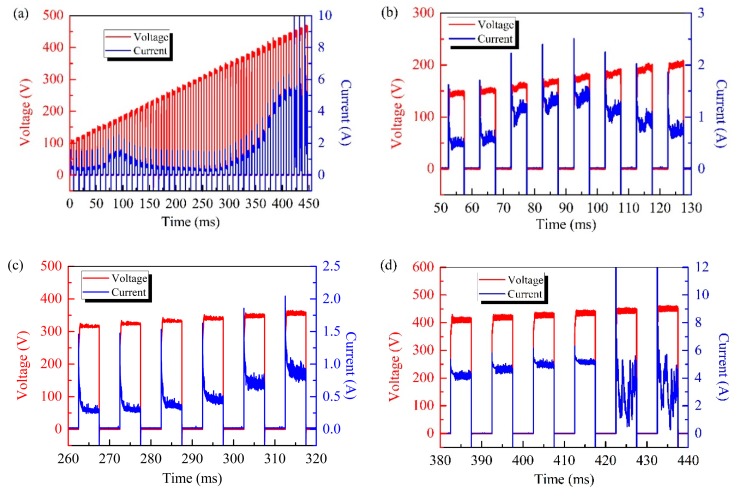
(**a**) Voltage and current waveforms of the initial test pulses and (**b**–**d**) partial waveforms.

Figure 2a shows that the current waveform changes by following a well-defined trend: When the voltage reached the corrosion potential of the substrate (Figure 2b, about 150 V), the current increased slightly due to the dissolution of the precursor film. Upon the growth of the oxide film, the plasma electrolytic oxidation system tended to stabilize and the current slowly dropped. When the voltage reached 330 V, the current abruptly increased (Figure 2c) and this might result in the dielectric breakdown of the oxide film. At this point, the current increased sharply when the voltage was further increased, implying that higher voltages led to more pronounced discharge events. Figure 2d shows the trend of the oscillating current for voltages higher than 450 V. In these conditions, powerful discharges might occur, causing destructive effects in the sample as reported in previous literature [6,11]. In this work, an initial *V_b_* equal to 330 V and a *V_u_* value of 450 V were chosen.

**Figure 3:**
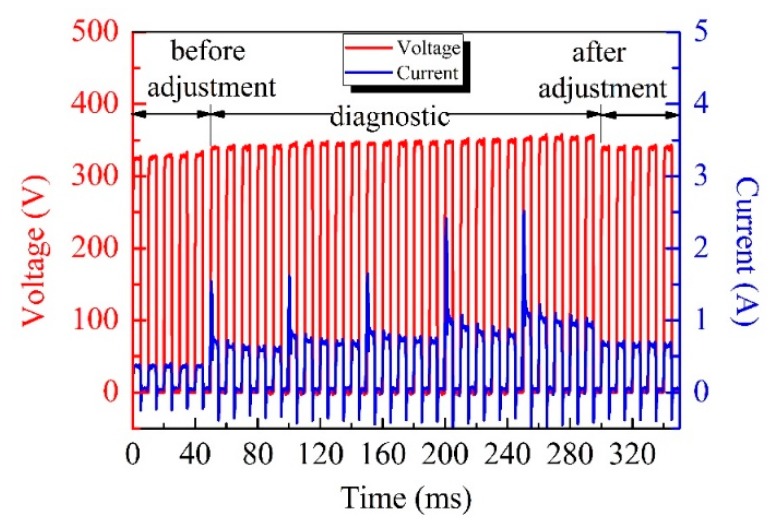
Voltage and current waveforms of pulses during a typical self-adaptive adjustment process.

Figure 3 depicts the waveforms of the pulses used in the test experiment and during the voltage adjustment process. When the value of the real-time feedback current was lower than half the value of *I_b_*, a new series of test pulses (330–350 V) was applied. According to the current waveforms of the test pulses, new values of *V_b_* (335 V) and *I_b_* (0.6 A) were determined and they correspond to the inflection point of the current curve. At this point, a new value of the amplitude (340 V) was assigned to the voltage pulses.

**Figure 4:**
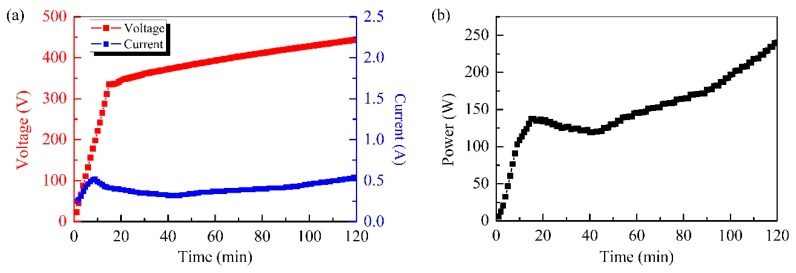
(**a**) Voltage–time curve, current–time curve, and (**b**) power–time curve during the self-adaptive plasma electrolytic oxidation processing of aluminum alloys.

Figure 4a shows the voltage and the current as a function of the measurement time: The voltage increased linearly in the 330–450V range and the current remained rather stable. These observations show that the current overshoot process, which occurs in the traditional voltage-control mode [6], was effectively suppressed. The current termination rule adopted in this work more effectively maintained a steady voltage in time, when compared to the more commonly used fixed-time methods. Figure 4b shows the output power curve, which was obtained experimentally: The power increased as a function of the processing time, reflecting that a higher pulse energy should be applied during the coating growth process to ensure the dielectric breakdown and a series of discharges.

### 3.2. Coating Microstructure

**Figure 5:**
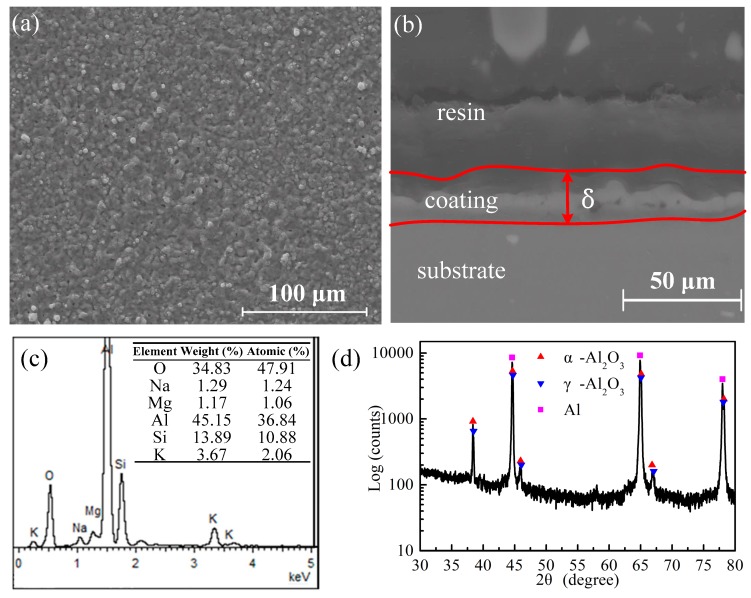
(**a**) Surface morphology, (**b**) cross-section, (**c**) chemical compositions, and (**d**) phases of the plasma electrolytic oxidation coatings.

The surface morphology of the coating exhibits (Figure 5a) regions with elongated open pores and lighter gray areas. Open pores are generally observed for short processing times or when a low-voltage amplitude is applied to low-thickness coatings [15]. The cross-sectional image of the sample (Figure 5b) reveals that the interfaces between the substrate and the pores and between the inner and the outer layer of the coatings were characterized by a wavy profile. The estimated thickness of the coating measures 23 μm and this value was lower than the previously reported ones (100 μm) [15,24]. Figure 5c shows that the coating was mostly composed of Al, O, and Si. Moreover, the phases of the coating were mainly composed of the α-Al_2_O_3_ and γ-Al_2_O_3_ (Figure 5d), which could enhance the coating microhardness and are formed under a soft-sparking regime.

### 3.3. Energy Consumption

The specific energy consumption (*Q_c_*) of the plasma electrolytic oxidation process can be calculated by using Equation (4):

(4) $$Q_{c}=\frac{P\cdot t}{S\cdot \delta }=\frac{1}{S\cdot \delta }\int_{0}^{t}u\left(t\right)\cdot i\left(t\right)dt,$$

where, *P* represents the power consumption, *t* is the total processing time, *S* corresponds to the superficial area of the sample, and *δ* is the coating thickness.

The value of *Q_c_* estimated by using Equation (4) is 1.8 kW h m^−2^ μm^−1^: This value is similar to that obtained in a previous study [24] (2.5–2.7 kW h m^−2^ μm^−1^) and considerably lower than other results (26.7 kW h m^−2^ μm^−1^) reported in the literature [28].

## 4. Discussion

The oxides of aluminum substrate have large band gaps. Such band gaps might be associated with an oxide structure with highly stable thermodynamic properties. It is well established that a dielectric breakdown commonly occurs across a thin oxide film located on a substrate [1]. Hence, an anodic oxidation process was performed before the plasma electrolytic oxidation processing of the sample, with the aim to promote discharge formation and enhance the dielectric breakdown strength. In order to ensure the electrons do not travel through the oxide film, the final voltage of the anodic oxidation process was set as 300 V, which is lower than the initial breakdown voltage.

The band gaps influence the electric field that tended to build up across the oxide film. A dielectric breakdown strength was normally expressed as a critical breakdown field, at which point, a discharge occurred. Hence, in order to assure enough dielectric breakdown strength, the applied voltage magnitude should be higher than the breakdown voltage. During the plasma electrolytic oxidation process, the electric fields were affected by the dielectric constant, which represents the capacity of the oxide to store electric charge. The dielectric constant increased with increasing thickness of the coating. Thus, the applied voltage should also be incremental. While the increasing voltage might show nonlinear behavior. Therefore, it is necessary to establish the relationship between the breakdown voltage and the coating thickness. The real-time pulse test technique provides a feasible approach to realize the identification of the feature information related to the coating growth.

The repeated formation of discharges on the surface of the sample is the key characteristic of a plasma electrolytic oxidation process. An individual event consisted of a complicated process such as micro-discharges, plasma channels, melting, evaporation, ejection, atomization, ionization, chemical reaction, cooling down, and overgrowth. According to previous research [1,5,10,15,20], the discharges change following a well-defined trend: The first spark, generating a discharge phenomenon, appeared by dielectric breakdown through a “weak site” in the anodic oxide film. The number of weak sites reduced with increasing thickness of the coating. With increasing voltage, the discharge color turned to orange–red, along with the emergence of more intense acoustic noise during the plasma electrolytic oxidation process. The individual discharges become less frequent but more intense when the increasing thickness of the coating, due to the reduced number of discharging sites through which the higher applied voltage needed to offer supplementary energy. These discharges have a strong tendency to occur repeatedly at particular locations—for example, they occur in ‘cascades’ that typically consist of hundreds of individual discharges [11]. Moreover, these discharges become more energetic and more dispersed—in terms of time and location—as the thickness increases. This is also verified by the evolving microstructure of the coating, particularly the pore content and architecture [20]. In a word, with an increasing applied voltage, the size of micropores, discharge channels, and overgrowth protrusions increased, which are mainly attributed to the different discharge energy supplied by high voltage. However, when the applied voltage is too high, negative effects on high-quality and good-performance coatings are observed. Hence, the discharge energy and coating microstructure are dependent on the electrical parameters of the process.

These results indicate that the discharge events, which occur during the self-adaptive plasma electrolytic oxidation process, mainly belong to the first three stages described in the literature [15]: Stage 1, a thin oxide film was formed and dielectric breakdown was observed; stage 2, many white sparks were evenly distributed on the entire surface of the sample; stage 3, the sparks were gradually replaced by more intense micro-discharges with yellow or orange appearance. All of the discharges mentioned above are maintained in the soft-sparking regime. When a low *V_u_* value is chosen, the discharges occur in the soft-sparking regime and this prevents the formation of cracks and other destructive effects on the coating, but this influences the choice of the coating thickness. The solution of such a compromise lies in the use of a novel soft-sparking regime based on the bipolar pulse mode, which occurs only when the ratio between the anodic and cathodic charge is lower than one [19,20,21,29].

It was estimated [32] that an individual discharge energy was ~1 mJ and that the conversion rate between discharge energy and resultant volume of the coating was ~10^13^ J m^−3^, which can also be expressed as about 3 kW h m^−2^ μm^−1^. The specific energy consumption of the self-adaptive plasma electrolytic oxidation process was only 1.8 kW h m^−2^ μm^−1^. Such improvement might be related to the use of the pre-anodized precursor films and the self-adaptive control of several process parameters, such as the voltage, the current, and the processing time. Recently, the precursor anodic porous films prepared by conventional anodizing were demonstrated to reduce the energy consumption and to increase the coating microhardness via promotion of the soft-sparking regime [24,25]. Meanwhile, the energy associated with each discharge may be optimally controlled via real-time precision adjustment of the process electrical parameters.

## 5. Conclusions

(1) A novel self-adaptive control method was used to prepare plasma electrolytic oxidation coatings on 6061 aluminum alloy samples.
(2) The feature information (*V_b_*, *I_b_*, *V_i_*, and *V_u_*) related to the coating growth was obtained from real-time feedback electrical signals generated via irregular test pulses. The process parameters (voltage, current, and processing time), which were applied during the coating preparation, were automatically adjusted according to the feature information.
(3) The coating produced via plasma electrolytic oxidation exhibited good uniformity and compactness, due to the extensive distribution of the small scattered ceramic particles. Moreover, no cracks and large pores were observed.
(4) The specific energy consumption of the self-adaptive plasma electrolytic oxidation process measured only 1.8 kW h m^−2^ μm^−1^.

## 6. Patents

Cao, B.; Yang, K.; Huang Z. Adaptive control method and system for plasma electrolytic oxidation process. *China ZL* 201410036861.5, 2016.

## Figures and Tables

**Table 1:** Parameters obtained during the test pulses experiment.

Type	Frequency (*f*, Hz)	Duty Cycle (*d*)	Number (*n*)	Growth (Δ*P*, V)	Basic Value (*U_e_*, V)
1	100	50%	45	8	100
2	100	50%	5 × 5 *	5	*V*_bn−1_

* In type 2, the step number of test pulses is equal to 5, the pulse number for the same step is equal to 5, and *V_b_*_n−1_ corresponds to the last breakdown voltage.
